# Clinical significance of the globulin‐to‐albumin ratio for prediction of postoperative survival in patients with colorectal cancer

**DOI:** 10.1002/ags3.12201

**Published:** 2018-08-29

**Authors:** Hiroyuki Hachiya, Mitsuru Ishizuka, Kazutoshi Takagi, Yoshimi Iwasaki, Norisuke Shibuya, Yusuke Nishi, Taku Aoki, Keiichi Kubota

**Affiliations:** ^1^ Second Department of Surgery Dokkyo Medical University Tochigi Japan

**Keywords:** biomarker, colorectal cancer, globulin‐to‐albumin ratio, inflammation‐based prognostic system, systemic inflammatory response

## Abstract

**Aim:**

A previous study has revealed that the albumin/globulin ratio (GAR) before treatment is a predictor of cancer‐specific survival in patients with colorectal cancer (CRC). The aim of the present study was to investigate the clinical significance of GAR for prediction of postoperative survival in patients with CRC.

**Methods:**

Nine hundred and forty‐one patients who had undergone elective CRC surgery were enrolled. Uni‐ and multivariate analysis models were performed to detect the clinical characteristics that were most closely associated with overall survival (OS). All recommended cutoff values were defined using receiver operating characteristic curve (ROC) analyses. Kaplan–Meier analysis was used to compare the OS curves between the high GAR (GAR > 0.83) and low GAR (GAR ≤ 0.83) groups.

**Results:**

Multivariate analysis using eight clinical characteristics selected by univariate analyses showed that the GAR was associated with OS (>0.83/≤0.83) (hazard ratio [HR], 1.979; 95% CI, 1.321‐2.966; *P* = 0.001) along with carcinoembryonic antigen (CEA; >8.7/≤8.7, ng/mL; HR, 2.319; 95% CI, 1.569‐3.428; *P* < 0.001), carbohydrate antigen 19‐9 (CA19‐9; >18.5/≤18.5, U/mL; HR, 1.727; 95% CI, 1.178‐2.532; *P* = 0.005), and the neutrophil‐to‐lymphocyte ratio (NLR; >2.9/≤2.9; HR, 2.132; 95% CI, 1.454‐3.126; *P* < 0.001), and the area under the ROC (AUROC) curve revealed that the GAR had the largest AUROC among these four clinical characteristics (GAR 0.711 > CEA 0.698 > CA19‐9 0.676 > NLR 0.635). A significant difference in OS was observed between patients with low GAR and those with high GAR (*P* < 0.001).

**Conclusion:**

Globulin‐to‐albumin ratio is a useful predictor of postoperative survival in patients with CRC.

## 1 INTRODUCTION

In Japan, colorectal cancer (CRC) is the second leading cause of cancer‐related death, accounting for approximately 50 000 deaths annually.^1^ CRC is also the third most common cancer and the fourth leading cause of cancer‐related death worldwide.^2^

The term “biomarker” commonly means a measurable serum protein substance whose concentration reflects the presence or degree of progression of a neoplasm. In the clinical field, prognostic biomarkers are often used for treatment decision‐making, because they are known to be useful predictors of outcome in cancer patients.^3^ In fact, the neutrophil‐to‐lymphocyte ratio (NLR),^4^ carcinoembryonic antigen (CEA),^5^ and carbohydrate antigen 19‐9 (CA19‐9)^5^ have been broadly used as prognostic biomarkers for patients with CRC. In particular, we have previously reported that the C‐reactive protein (CRP)‐to‐albumin ratio (CAR)^6^ and the Glasgow Prognostic Score (GPS)^7^ are also useful for prediction of postoperative outcome in patients with CRC.

Cancer cells and other cells or tissues at sites of inflammation produce and secrete excessive amounts of inflammatory cytokines. Interleukin‐6 (IL‐6) is a typical inflammatory cytokine, and its level is known to be increased in patients with cancer.^8^ IL‐6 not only induces differentiation of B‐lymphocytes into plasma cells to produce immunoglobulins, but also decreases albumin production in the liver.^9^

Recently, this mechanism has been the focus of attention in patients with CRC, and the albumin/globulin ratio is reported to be useful for prognostication in such patients.^10^ We have also reported that the globulin‐to‐albumin ratio (GAR) is a useful predictor of postoperative survival in patients with hepatocellular carcinoma (HCC) undergoing potentially curative liver resection.^11^ Because the GAR is the ratio of albumin relative to globulin, it is less affected by measurement variability and can be determined as easily as the NLR. However, the first study of GAR in CRC patients did not determine the ideal cutoff value, and therefore, further refinement of this value would be of potential clinical utility.^10^

Using a clinical database at a single institution, this study was conducted to determine whether the preoperative GAR would be useful for prognostication in CRC patients.

## 2 METHODS

A retrospective review was performed using a database of patients who had undergone elective surgery for CRC. All of the procedures had been performed by the same surgical team at the Department of Gastroenterological Surgery, Dokkyo Medical University Hospital, between June 2006 and March 2016. During this period, 1002 patients who had undergone surgery were enrolled.

No patients had clinical evidence of infection or other inflammatory conditions, and none had received preoperative chemotherapy or irradiation. Therefore, we excluded 61 patients with colon perforation and 47 patients with small or large bowel obstruction requiring decompression (22 patients received transnasal or transanal ileus tube insertion, five patients received colonic stent insertion, and 20 patients underwent colostomy). In addition, 10 patients who received preoperative chemotherapy were excluded. Routine laboratory measurements including the serum levels of total protein, albumin, and tumor markers such as CEA^12^ (upper physiological value: 5 ng/mL) and CA19‐9^12^ (upper physiological value: 37 U/mL) were carried out on the day of admission. This study complied with the standards of the Declaration of Helsinki and current ethical guidelines and was approved by our institutional ethics board.

The cutoff values for various clinical characteristics were determined using receiver operating characteristic (ROC) curve analyses. The recommended cutoff values were based on the most prominent point on the ROC curve for “sensitivity” and “1 – specificity,” respectively. Then, the ideal cutoff values were defined using the Youden index maximum [sensitivity − (1 − specificity)].^13^ The area under the ROC (AUROC) curve was also calculated.

For example, the optimal cutoff value for the GAR corresponded to the point on the ROC curve showing the best sensitivity (0.767) and specificity (0.549), respectively. For these parameters, the AUROC was 0.711 and the optimal cutoff value of 0.831. The categorical clinical characteristics were examined in two patient groups: low GAR (GAR ≤ 0.83) and high GAR (GAR > 0.83). The cutoff values for other clinical characteristics were calculated in the same way.

Univariate analysis was performed to evaluate preoperative clinical characteristics including age (>60/≤60, year), gender (female/male), tumor site (colon/rectum), number of tumors (≥2/1), maximum tumor diameter (>50/≤50, mm), tumor type (3, 4, 5/0, 1, 2), pathology (others/well or moderately differentiated), depth of tumor (Tis, T1, T2/T3, T4), lymph node metastasis (N0/N1, 2), white blood cell (WBC) count (>6.6/≤6.6, ×10^3^/mm^3^), platelet count (>27/≤27, ×10^4^/mm^3^), CEA (>8.7/≤8.7, ng/mL), CA19‐9 (>18.5/≤18.5, U/mL), NLR (>2.9/≤2.9), and GAR (>0.83/≤0.83) to select those that were useful for prediction of overall survival (OS). All cutoff values were defined using ROC analyses, except for gender, tumor site, number of tumors, tumor type, and pathology.

Inflammation‐based prognostic system consists of not only protein‐based system but also blood cell‐based system such as NLR and thrombocytosis. Therefore, WBC and platelet are also added to the univariate analysis in the study. Multivariate analysis was performed using the clinical characteristics selected by univariate analysis with a *P*‐value of <0.05.

### 2.1 Definition of inflammation‐based prognostic systems

The GPS was calculated as follows: Patients with both an elevated level of CRP (>1.0 mg/dL) and hypoalbuminemia (albumin <3.5 g/dL) were allocated a score of 2, and patients showing one or neither of these blood chemistry abnormalities were allocated a score of 1 or 0, respectively.^14^

The NLR was calculated as follows: NLR = neutrophil ratio (%) (or number of neutrophils)/lymphocyte ratio (%) (or number of lymphocytes).^4^

The GAR was calculated as follows: GAR = serum globulin level (mg/dL) [serum total protein level (g/dL) − serum albumin level (g/dL)]/serum albumin level (g/dL).^11^

### 2.2 Definition of operative curability

On the basis of the Japanese Classification of Colorectal Carcinoma (Japanese Society for Cancer of the Colon and Rectum, Second English Edition),^15^ residual tumor is diagnosed as R2; no residual tumor as R0; no residual tumor, but tumor suspected at the resection margin as R1; and macroscopically evident residual tumor. On the basis of this definition, operative curability is defined as follows: curability A (Cur A), R0 at stage I, II, or III; curability B (Cur B), R0 at stage IV or R1 at any stage; and curability C (Cur C), R2 at any stage.^16^

### 2.3 Administration of chemotherapy

Most stage IV patients undergoing surgery were considered for postoperative chemotherapy. Because recent chemotherapy regimens such as FOLFIRI^17^ and FOLFOX^18^ were introduced in our department in January 2005, patients who had undergone surgery before January 2005 had been administered oral anticancer drugs based on 5‐fluorouracil postoperatively. In addition, recent antitumor antibody agents such as bevacizumab,^19^ cetuximab^20^, and panizummab^21^ were started in our department in December 2007, February 2009, and March 2011, respectively.

Similarly, most stage III patients undergoing surgery were considered for postoperative chemotherapy. Because introduction of recent chemotherapy regimens such as capecitabine^22^ for colon cancer and UFT+leucovorin^23^ for rectal cancer was started in our department in October 2008, patients who had undergone surgery before October 2008 were administered the same drugs as those for stage IV patients.

In addition, most stage II patients undergoing surgery were not considered for postoperative chemotherapy.^24^

### 2.4 Statistical analysis

Data are presented as mean ± SD (standard deviation). Differences among the groups were analyzed using the χ^2^ test and Mann–Whitney *U*‐test. Hazard ratio (HR) with 95% CI was calculated by univariate or multivariate analysis using the Cox proportional hazards model. Multivariate analysis was performed using clinical characteristics with *P* < 0.05 selected in the univariate analysis to assess those most closely related to OS.

Kaplan–Meier analysis and log rank test were used to compare the survival curves for the two groups. Deaths prior to 31 December 2014 were included in this analysis.

Statistical analyses were performed using the IBM SPSS statistics version 22.0 software package for Windows (IBM Co., New York, NY, USA) at a significance level of *P* < 0.05.

## 3 RESULTS

A total of 941 patients were enrolled (male:female = 581:360). There were 473 patients with low GAR (GAR ≤ 0.83) and 468 with high GAR (GAR > 0.83).

Table 1 shows the distribution of the categorical clinical background characteristics of the studied patients in the two GAR groups. There were significant differences between the two groups in age (≤60/>60, year), tumor site (colon/rectum), number of tumors (1/≥2), maximum tumor diameter (≤50/>50, mm), tumor type (3, 4, 5/0, 1, 2), pathology (well or moderately differentiated/others), lymphatic invasion (ly0/ly1, 2, 3), venous invasion (v0/v1, 2, 3), WBC count (≤6.6/>6.6, ×10^3^/mm^3^), platelet count (≤27/>27, ×10^4^/mm^3^), CEA (≤8.7/>8.7, ng/mL), CA19‐9 (≤18.5/>18.5, U/mL), NLR (≤2.9/>2.9), GPS (0/1/2), type of surgery (open/laparoscopic), operative curability (A/B/C), depth of tumor (Tis, T1, T2/T3, T4), and stage (0/I/II/III/IV; χ^2^ test).

**Table 1:** Relationships between the categorical clinical characteristics and GAR in patients with CRC

Variable	GAR ≤ 0.83 (*n* = 473)	GAR > 0.83 (*n* = 468)	*P*‐value
Age (years)			
≤60	136	86	
>60	337	382	<**0.001**
Gender			
Male	291	290	
Female	182	178	0.893
Tumor site			
Colon	290	318	
Rectum	183	150	**0.035**
Number of tumors			
1	434	401	
≥2	36	53	
Undetermined	3	14	**0.003**
Maximum tumor diameter (mm)			
≤50	357	211	
>50	99	191	
Undetermined	17	66	<**0.001**
Tumor type			
0, 1, 2	400	330	
3, 4, 5	62	90	
Undetermined	11	48	<**0.001**
Pathology			
Well or moderately	435	405	
Others	31	36	
Undetermined	7	27	**0.001**
Lymphatic invasion			
Absence	153	101	
Presence	305	310	
Undetermined	15	57	<**0.001**
Venous invasion			
Absence	132	87	
Presence	327	324	
Undetermined	14	57	<**0.001**
WBC count (×10^3^/mm^3^)			
≤6.6	287	203	
>6.6	186	265	<**0.001**
Platelet count (×10^4^/mm^3^)			
≤27	295	216	
>27	178	252	<**0.001**
CEA (ng/mL)			
≤8.7	373	280	
>8.7	100	188	<**0.001**
CA19‐9			
≤18.5	350	282	
>18.5	123	184	
Undetermined	0	2	<**0.001**
NLR			
≤2.9	177	250	
>2.9	296	218	<**0.001**
GPS			
0	429	138	
1	41	168	
2	3	162	<**0.001**
Surgery			
Open	218	378	
Laparoscopic	239	66	
Conversion to open surgery	14	22	
Others	2	2	<**0.001**
Operative curability			
A	402	284	
B	31	48	
C	40	136	<**0.001**
Depth of tumor			
Tis, T1, T2	154	77	
T3, T4	313	353	
Undetermined	6	38	<**0.001**
Lymph node metastasis			
N0	266	217	
N1, 2	197	187	
Undetermined	10	64	0.274
Stage			
0	22	14	
I	111	56	
II	123	122	
III	151	98	
IV	63	155	
Undetermined	3	23	<**0.001**

CA19‐9, carbohydrate antigen 19‐9; CEA, carcinoembryonic antigen; CRC, colorectal cancer; GAR, globulin‐to‐albumin ratio; GPS, Glasgow Prognostic Score; NLR, neutrophil‐to‐lymphocyte ratio; WBC, white blood cell. Bold values indicate a significant difference.

Table 2 shows the continuous clinicolaboratory characteristics of the two GAR groups. There were significant intergroup differences in age (year), number of tumors, maximum tumor diameter (mm), CRP (mg/dL), albumin (g/dL), globulin (g/dL), WBC count (×10^3^/mm^3^), platelet count (×10^4^/mm^3^), CEA (ng/mL), CA19‐9 (U/mL), and NLR (Mann–Whitney *U*‐test).

**Table 2:** Relationships between the continuous clinicolaboratory characteristics and GAR in patients with CRC

Variable	GAR ≤ 0.83 (*n* = 473)	GAR > 0.83 (*n* = 468)	*P*‐value
Age (years)	66 ± 12	71 ± 11	<**0.001**
Number of tumors	1.09 ± 0.33	1.14 ± 0.42	**0.036**
Maximum tumor diameter (cm)	3.9 ± 1.9	5.4 ± 2.5	<**0.001**
CRP (mg/dL)	0.3 ± 0.9	2.6 ± 4.8	<**0.001**
WBC count (×10^3^/mm^3^)	6.6 ± 2.3	7.7 ± 3.6	<**0.001**
Platelet count (×10^4^/mm^3^)	26 ± 7	30 ± 12	<**0.001**
CEA (ng/mL)	26 ± 166	139 ± 859	<**0.001**
CA19‐9 (U/mL)	106 ± 722	418 ± 1766	<**0.001**
NLR	3.0 ± 3.2	4.4 ± 4.7	<**0.001**
Observed period (d)	788 ± 733	810 ± 805	0.505

CA19‐9, carbohydrate antigen 19‐9; CEA, carcinoembryonic antigen; CRC, colorectal cancer; CRP, C‐reactive protein; GAR, globulin‐to‐albumin ratio; NLR, neutrophil‐to‐lymphocyte ratio; WBC, white blood cell.

Mean ± SD, Mann–Whitney *U*‐test. Bold values indicate a significant difference.

During the observation period, 160 patients died, among whom 125 died of cancer‐related disease. Univariate and multivariate analyses were performed to evaluate the relationship between clinical characteristics and OS.

The results of univariate analyses demonstrated that tumor type (3, 4, 5/0, 1, 2), pathology (others/well or moderately differentiated), depth of tumor (Tis, T1, T2/T3, T4), lymph node metastasis (N0/N1, 2), WBC count (>6.6/≤6.6, ×10^3^/mm^3^), platelet count (>27/≤27, ×10^4^/mm^3^), CEA (>8.7/≤8.7, ng/mL), CA19‐9 (>18.5/≤18.5, U/mL), NLR (>2.9/≤2.9), and GAR (>0.83/≤0.83) were associated with OS (Table 3).

**Table 3:** Univariate analyses in relation to overall survival

Variable	*P*‐value	Hazard ratio	95% CI
Age (>60/≤60, years)	0.641	1.088	0.763‐1.553
Gender (Female/Male)	0.993	1.001	0.728‐1.378
Tumor site (Rectum/Colon)	0.997	1.001	0.724‐1.384
Number of tumors (≥2/1)	0.318	0.731	0.396‐1.351
Maximum tumor diameter (>50/≤50, mm)	0.087	1.358	0.957‐1.927
Tumor type (3, 4, 5/0, 1, 2)	**<0.001**	2.142	1.456‐3.152
Pathology (others/well or moderately)	**0.002**	2.168	1.323‐3.553
Depth of tumor (Tis, T1, T2/T3, T4)	**<0.001**	3.407	1.732‐6.705
Lymph node metastasis (N0/N1, 2)	**<0.001**	1.976	1.343‐2.907
WBC count (>6.6/≤6.6, ×10^3^/mm^3^)	**<0.001**	1.971	1.430‐2.716
Platelet count (>27/≤27, ×10^4^/mm^3^)	**0.001**	1.724	1.260‐2.361
CEA (>8.7/≤8.7, ng/mL)	**<0.001**	3.935	2.869‐5.396
CA19‐9 (>18.5/≤18.5, U/mL)	**<0.001**	2.974	2.168‐4.080
NLR (>2.9/≤2.9)	**<0.001**	3.001	2.162‐4.166
GAR (>0.83/≤0.83)	**<0.001**	2.883	2.020‐4.115

95% CI, 95% confidence interval; CA19‐9, carbohydrate antigen 19‐9; CEA, carcinoembryonic antigen; GAR, globulin‐to‐albumin ratio; NLR, neutrophil‐to‐lymphocyte ratio; WBC, white blood cell. Bold values indicate a significant difference.

Multivariate analysis using the above selected clinical characteristics disclosed that GAR (>0.83/≤0.83) was associated with OS (HR, 1.979; 95% CI, 1.321‐2.966; *P* = 0.001) along with CEA (>8.7/≤8.7, ng/mL; HR, 2.319; 95% CI, 1.569‐3.428; *P* < 0.001), CA19‐9 (>18.5/≤18.5, U/mL; HR, 1.727; 95% CI, 1.178‐2.532; *P* = 0.005), and NLR (>2.9/≤2.9; HR, 2.132; 95% CI, 1.454‐3.126; *P* < 0.001; Table 4).

**Table 4:** Multivariate analysis in relation to overall survival

Variable	*P*‐value	Hazard ratio	95% CI
Tumor type (3, 4, 5/0, 1, 2)	0.446	1.172	0.780‐1.761
Pathology (others/well or moderately)	0.063	1.716	0.971‐3.031
WBC count (>6.6/≤6.6, ×10^3^/mm^3^)	0.875	0.970	0.663‐1.419
Platelet count (>27/≤27, ×10^4^/mm^3^)	0.928	1.017	0.710‐1.457
CEA (>8.7/≤8.7, ng/mL)	**<0.001**	2.319	1.569‐3.428
CA19‐9 (>18.5/≤18.5, U/mL)	**0.005**	1.727	1.178‐2.532
NLR (>2.9/≤2.9)	**<0.001**	2.132	1.454‐3.126
GAR (>0.83/≤0.83)	**0.001**	1.979	1.321‐2.966

95% CI, 95% confidence interval; CA19‐9, carbohydrate antigen 19‐9; CEA, carcinoembryonic antigen; GAR, globulin‐to‐albumin ratio; NLR, neutrophil‐to‐lymphocyte ratio; WBC, white blood cell. Bold values indicate a significant difference.

However, the results of AUROC analysis revealed that the GAR had the largest AUROC among these four clinical characteristics as follows: GAR 0.711 > CEA 0.698 > CA19‐9 0.676 > NLR 0.635.

The median and maximum follow‐up periods for survivors were 559 and 3389 days, respectively, and the mean survival period was 799 ± 769 days (mean ± SD).

There was no significant difference during the observed period between patients with low GAR (≤0.83; 788 ± 733 days) and patients with high GAR (>0.83; 810 ± 805 days; *P* = 0.505, Mann–Whitney *U*‐test; Table 2).

**Figure 1:**
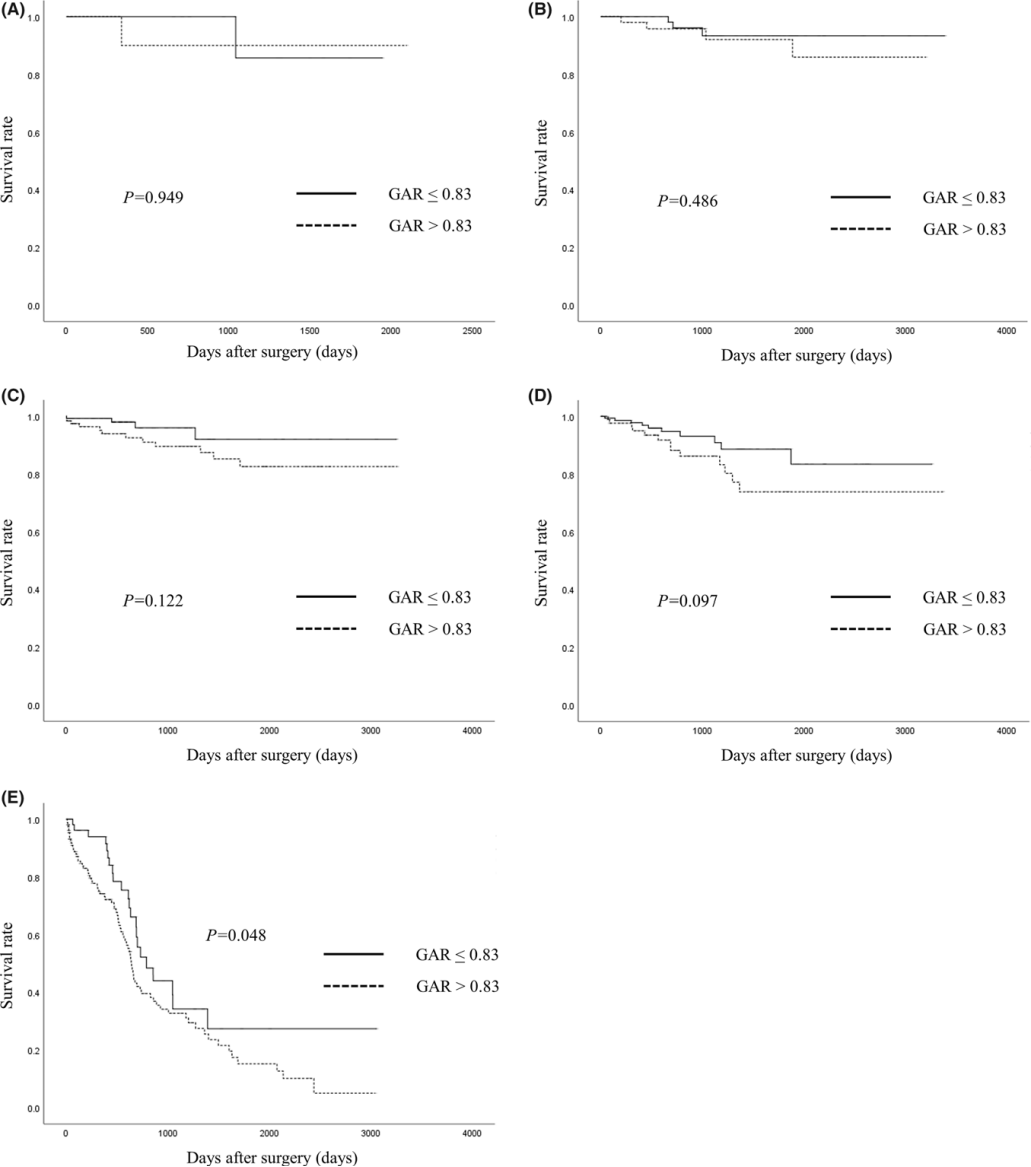
A, Relationship between the globulin‐to‐albumin ratio (GAR) and overall survival (OS) after surgery for stage 0 colorectal cancer (CRC) patients. B, Relationship between the GAR and OS after surgery for stage I CRC patients. C, Relationship between the GAR and OS after surgery for stage II CRC patients. D, Relationship between the GAR and OS after surgery for stage III CRC patients. E, Relationship between the GAR and OS after surgery for stage IV CRC patients

**Figure 2:**
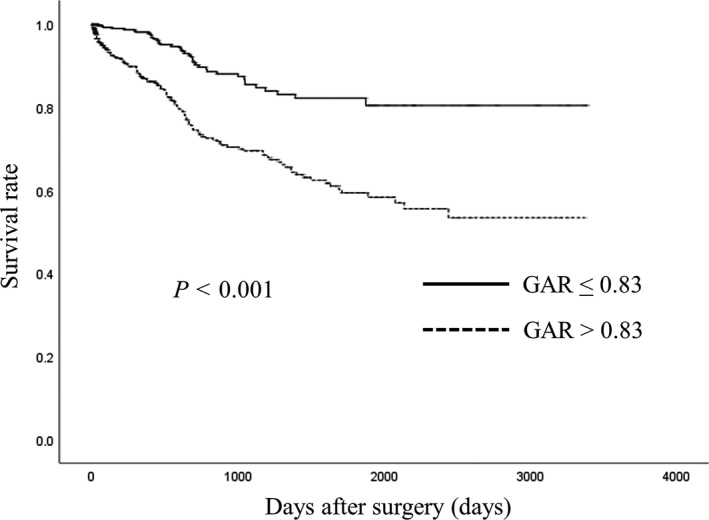
Relationship between the globulin‐to‐albumin ratio and overall survival after surgery for all stage colorectal cancer (CRC) patients

Although Kaplan–Meier analyses and log rank tests demonstrated that there were no significant differences between the two groups of stages 0, I, II, and III (GAR ≤ 0.8 vs GAR > 0.8) in OS (Figure 1A–D), there was a significant difference between the two groups of stage IV (GAR ≤ 0.8 vs GAR > 0.8) in OS (Figure 1E). There was a significant difference between patients with low GAR (≤0.83) and patients with high GAR (>0.83) of all stages in OS (*P* < 0.001). Patients with high GAR (>0.83) showed poorer OS than those with low GAR (≤0.83; Figure 2).

## 4 DISCUSSION

Advances in laparoscopic surgery and development of novel chemotherapies are improving the treatment of CRC. In recent years, “precision medicine” has also played a crucial role in maximizing the effects of therapy and minimizing side effects in individual patients. In addition to general medical information, it is important to explore the individual genetic backgrounds, physiological condition, and disease state of patients using biomarkers, and such information is effective for selection of appropriate treatment for each patient.

It has been reported that persistent chronic inflammation is involved in cancer development, progression, and metastasis.^25^ Therefore, such inflammation is associated with hypermetabolism, weight loss, and anorexia in cancer patients.^26^ In fact, acute‐phase proteins such as CRP, which is an important marker of systemic inflammatory response (SIR), is induced by inflammatory cytokines.^27^ Tumor necrosis factor‐α (TNF‐α), interleukin‐10 (IL‐10), and IL‐6 are also inflammatory cytokines that are well known to be involved in various types of cancer.^25^ Among them, IL‐6 is produced by a variety of cells including T cells, B cells, fibroblasts, monocytes, endothelial cells, and mesangial cells.^28^ IL‐6 is associated with the development and progression of CRC.^29^ Recent studies have shown that the IL‐6 level is significantly increased in the tumor microenvironment and that tumor proliferation is suppressed by anti‐IL‐6 receptor antibody.^30^ In fact, the transmembrane IL‐6 receptor is expressed on the cell surface of hepatocytes.^31^ When IL‐6 acts on hepatocytes, there is an increase in gene transcription rate of acute‐phase proteins such as CRP, and the rate and synthesis of albumin mRNA transcription compensates decreases.^32^ Similarly, when IL‐6 acts on B cells, it induces their differentiation into plasma cells, which produce antibody and upregulate immunoglobulin production.^7^

Previous studies have also shown that the preoperative albumin/globulin ratio is an independent prognostic factor in CRC patients.^10^ Serum proteins are divided into two groups: albumin and globulins. Serum albumin constitutes approximately 60% of the total protein,^7^ being produced in the liver and functioning to maintain osmotic pressure and the transport of various metabolites. Albumin also has an antioxidant effect against carcinogens (e.g., nitrosamines and aflatoxins) by stabilizing cell proliferation and deoxyribonucleic acid (DNA) replication.^33^ Laursen et al.^34^ have reported that the growth of a human breast cancer cell line was inhibited by albumin. Other researchers have reported that some cancer patients with hypoalbuminemia have shorter OS and higher recurrence rates.^35^ On the other hand, globulins are divided into four groups according to their electrophoretic profiles: alpha 1, alpha 2, beta, and gamma globulins. Among them, gamma globulin, also called immunoglobulin, is an antibody that plays an extremely important role in the immune system against disease.^36^ It is known that the globulin level increases with chronic inflammation due to the activity of inflammatory cytokines.^37^ Because the serum levels of albumin and globulin are affected by many factors such as stress, liver failure, dehydration, and edema, they also show measurement variability. However, because the GAR is a ratio rather than an absolute value, it is less affected by measurement variability than the serum levels of albumin and globulin separately. In fact, we have reported that even if patients have liver dysfunction, the GAR would be rarely affected by such condition and could predict the postoperative survival of such patients.^11^

The present multivariate analysis revealed four prognostic biomarkers: GAR, NLR, CEA, and CA19‐9. However, CEA and CA19‐9 may show normal levels even in patients with advanced cancer. In fact, it has been reported that approximately 73% of CRC patients who underwent surgery had normal preoperative serum CEA levels^38^ and 84% had normal CA19‐9 levels.^39^ On the other hand, GAR is based on protein components and NLR is based on cell components, and they are less likely to reflect tumor characteristics as both are indicators of inflammation and immune status in individual patients. The AUROC of the four biomarkers was 0.771 for GAR, 0.698 for CEA, 0.676 for CA19‐9, and 0.635 for NLR, with GAR having the largest AUROC and demonstrating the closest relationship with outcome in patients with CRC. The cutoff value of GAR determined in our department was 0.93 for HCC and 0.83 for CRC.^11^ Because it is difficult to define the ideal cutoff value for GAR because of variation of organs, future studies will need to examine the utility of GAR as a prognostic biomarker. The present study showed superiority over the previous one^10^ as its sample size was almost 2‐fold larger, and the data were examined in terms of cutoff values. In addition, GAR is less expensive to determine than commonly used tumor markers, and can be repeatedly measured, making it not only versatile but also universally applicable.

In conclusion, the GAR can be used as an easy, cheap, objective, and noninvasive biomarker for prognostication of CRC patients undergoing surgery and is useful for prediction of postoperative survival.

## DISCLOSURE

Conflict of Interest: All authors have no conflict of interests.

Author Contribution: All authors meet all of the criteria for the definition of authorship: substantial contributions to the conception or design of the work, or the acquisition, analysis, or interpretation of data for the work; drafting the work or revising it critically for important intellectual content; final approval of the version to be published; and agreement to be accountable for all aspects of the work in ensuring that questions related to the accuracy or integrity of any part of the work are appropriately investigated and resolved.
